# Impact of Optimal Timing of Intake of Multi-Ingredient Performance Supplements on Sports Performance, Muscular Damage, and Hormonal Behavior across a Ten-Week Training Camp in Elite Cyclists: A Randomized Clinical Trial

**DOI:** 10.3390/nu13113746

**Published:** 2021-10-23

**Authors:** Diego Fernández-Lázaro, Juan Mielgo-Ayuso, Miguel del Valle Soto, David P. Adams, Eduardo Gutiérrez-Abejón, Jesús Seco-Calvo

**Affiliations:** 1 Department of Cellular Biology, Histology and Pharmacology, Faculty of Health Sciences, University of Valladolid, Campus of Soria, 42003 Soria, Spain; 2 Neurobiology Research Group, Faculty of Medicine, University of Valladolid, 47005 Valladolid, Spain; 3 Department of Health Sciences, Faculty of Health Sciences, University of Burgos, 09001 Burgos, Spain; 4 Department of Morphology and Cell Biology, University of Oviedo, Health Research Institute of the Principality of Asturias (ISPA), 33003 Oviedo, Spain; miva@uniovi.es; 5 Dual Enrollment Program, Point University, Savannah, GA 31419, USA; David.Adams@point.edu; 6 Pharmacological Big Data Laboratory, University of Valladolid, 47005 Valladolid, Spain; egutierreza@saludcastillayleon.es; 7 Technical Direction of Pharmaceutical Assistance, Regional Health Management of Castilla y León, 47005 Valladolid, Spain; 8 Physiotherapy Department, Institute of Biomedicine (IBIOMED), University of Leon, Visiting Researcher of Basque Country University, Campus de Vegazana, 24071 Leon, Spain; dr.seco.jesus@gmail.com

**Keywords:** supplementation, timing, amino acids, hormones, muscle recovery, sport performance

## Abstract

Multi-ingredient performance supplements (MIPS), ingested pre- or post-workout, have been shown to increase physiological level effects and integrated metabolic response on exercise. The purpose of this study was to determine the efficacy of pre-and post-training supplementation with its own MIPS, associated with CHO (1 g·kg^−1^) plus protein (0.3 g·kg^−1^) on exercise-related benchmarks across a training camp for elite cyclists. Thirty elite male cyclists participated in a randomized non-placebo-controlled trial for ten weeks assigned to one of three groups (*n* = 10 each): a control group treated with CHO plus protein after training (CG); a group treated with MIPS before training and a CHO plus protein after training, (PRE-MIPS); a group treated with CHO plus protein plus MIPS after training, (POST-MIPS). Performance parameters included (VO_2_max, peak; median and minimum power (W) and fatigue index (%)); hormonal response (Cortisol; Testosterone; and Testosterone/Cortisol ratio); and muscle biomarkers (Creatine kinase (CK), Lactate dehydrogenase (LDH), and Myoglobin (Mb)) were assessed. MIPS administered before or after training (*p* ≤ 0.05) was significantly influential in attenuating CK, LDH, and MB; stimulating T response and modulating C; and improved on all markers of exercise performance. These responses were greater when MIPS was administered post-workout.

## 1. Introduction

Elite cycling is one of the most physically demanding sports. It combines extremes of exercise duration and intensity [1]. Professional cyclists usually undergo strenuous periods of training before three-week races like the *Giro d’Italia*, *Tour de France,* and *Vuelta a España* [2]. Elite cyclists often participate in training camps to enhance training adaptation at specific times in the season in preparation for a specific event or competition [3]. The nature of training camps are extremely demanding in terms of physiology for improving aerobic or anaerobic capacity [2]. The physiological effect of training depends on the quality and quantity of exercise performed [4]. In training camps, elite cycling increases the exercise’s volume and intensity with strenuous training loads [5]. In conjunction with inadequate recovery, extreme high training loads can lead to a state of physiological overtraining characterized by changes in the athletes’ general health and physical performance [6]. As such, intermittent recovery periods are essential in these training camps [7]. In this way, cyclists can implement methods that protect their health, allowing them to perform full training, and improve competition results. These methods of recovery enhancement and improved sports performance are essential in settings where athletes compete for several consecutive days and could be based on products that help meet increased energy and nutrient needs, supply fluids and elements lost during physical activity [8].

There is generally less than 20 h (h) between their daily training sessions. It becomes necessary, therefore, to guarantee an adequate intake of carbohydrates (CHO) and protein during within the first four hours of the recovery period, not only to replenish hepatic glycogen reserves, but also to maximize connective repair tissue and stimulate muscle recovery [9,10]. CHO and protein supplementation (1 g·kg^−1^ and 0.3 g·kg^−1^) after each exercise session facilitates recovery from strenuous training in athletes [6,7]. The total daily and post-workout intake of CHO, protein, and fat suggest [6,7]: (i) Dietary protein intake is necessary to support metabolic adaptation, repair remodeling, and for protein turnover generally ranges from 1.2 to 2.0 g/kg/day. A higher intake may be indicated for short periods during intensive training or when energy intake is reduced; (ii) With respect to recommendations that the proportion of energy from fat be limited to less than 10% and that sources of essential fatty acids be included to meet adequate intake recommendations. Fat intake by athletes should be in line with public health guidelines and should be individualized based on training level and body composition goals; (iii) An intake of 1.5 g CHO/kg body weight within 30 min after exercise and 5–10 g CHO/kg body weight daily has been shown to increase the rate of muscle glycogen resynthesis. Although it should be considered that all these recommendations cover most training regimens and allow for flexible adjustments based on training and experience. It becomes essential, then, to prioritize the recovery period and educate athletes about the importance of following CHO and protein guidelines following strenuous exercise sessions [11].

In settings such as training camps or three-week races where training demands increase, however, additional nutritional ergogenic aids may provide additional support for athletes to better withstand the training plan and to improve performance in competition [12]. Performance-enhancing dietary supplements have gained popularity among athletes, and between 37 and 89% of athletes report use of such products [13]. Nutritional ergogenic aids are consumed before and after training to improve overall performance [6]. At present, a novel class of dietary supplements, multi-ingredient performance supplements (MIPS), have become increasingly popular. MIPS include branched chain amino acids (BCAAs), creatine monohydrate, L-arginine, L-glutamine, L-taurine, caffeine, β-alanine, L-citrulline, L-carnitine, and black pepper fruit extract (piperine), and are intended for ingestion prior to or after exercise sessions with the intention of inducing an acute ergogenic effect [14]. It is one of the two types of MIPS. The second MIPS is composed of strictly ergogenic substances that provide metabolic support by supplementing ingredients essential for bioenergetic or anabolic processes that allow attenuating skeletal muscle damage or regulating hormonal behavior [15]. Some dietary supplements have been used in dual combinations and have demonstrated a synergistic effect by increasing sports performance, testosterone concentration, and testosterone/cortisol ratio. In addition, these dietary supplements showed synergism in decreasing muscle damage and cortisol levels. This could prove beneficial for post-training metabolic/physiological adjustments [14,16,17]. Nonetheless, a lack of studies exist concerning the combined effect of MIPS on cyclists. Competitive cycling produces quite different metabolic/physiological demands associated with distinct routes and other types of efforts such as aerobic on flat surfaces or anaerobic on inclines and while performing sprints [18].

Another critical factor to consider is the dose and timing of supplementation. Numerous studies on nutrient timing have examined this issue [19,20,21,22,23]. Athletes can use MIPS either before [7,12,22] or immediately after a workout [24]; however few studies have studied the effect of MIPS in both situations on resistance training [14,25]. The effectiveness of MIPS before and after each session of an intense ten- week training camp for cyclists from three professional teams before *Vuelta a España* has not been investigated. Therefore, this study aimed to determine the efficacy of pre-and post-supplementation with MIPS associated with CHO ((1 g·kg^−1^) maltodextrin, maltose, and fructose) plus whey protein isolate (0.3 g·kg^−1^) on skeletal muscle biomarkers, hormonal response, and sports performance in elite cyclists.

## 2. Material and Methods

### 2.1. Experimental Desing

In 2020, thirty elite male cyclists (*n* = 30) who were members of different professional cycling teams participated in this randomized, non-placebo-controlled trial to analyze the effects of the combination of oral CHO, proteins, MIPS supplementation for 10 weeks on muscle damage levels (creatine kinase (CK), lactate dehydrogenase (LDH) and myoglobin (Mb)), hormonal status as measured by the catabolic hormone cortisol, anabolic hormone testosterone, and testosterone/cortisol ratio. Aerobic (maximum oxygen consumption (VO2max)) and anaerobic (Wingate test) sports performance were also analyzed.

All athletes performed the same training sessions during the training-camp (pre- competitive period) that consisted of 5–6 h per day, 6 days per week for 10 weeks. Our dietitian-nutritionist also developed an individual diet based on pre-established energy and macronutrient guidelines for adequate athletic performance and each participant’s training volume and training load [26]. A pre-study medical exam was done to ensure that participants did not have any preexisting illnesses or injuries. Participants also self-reported they did not use illegal drugs (stimulants, blood derivatives, anabolic agents) or take medications (tramadol) or other ergogenic products that could affect the analytical variables and/or anthropometrics under study. All participants were fully informed of all aspects of the study and signed an informed consent statement. This research was designed according to the Declaration of Helsinki (2008), with the Fortaleza Update (2013) (World Medical Association, 2013). The Ethics Committee on Human Research approved it at the European University of Madrid, Madrid, Spain, with the internal number CIPI/20/107.

### 2.2. Experimental Protocol and Assessment Plan

Participants were randomly assigned to 3 groups using a stratified block design. An independent statistician generated the randomization sequence:

Control group (CG) treated with 1 g·kg^−1^ CHO plus whey protein isolate (0.3 g·kg^−1^) in the form of a recovery shake (mixing with ~200 mL of plain water) within half an hour after completion of exercise (CG, *n* = 10). In addition, ~150 mL the placebo-flavored water (placebo plus flavors only) in the 30 min pre- and 30 min post-exercise.

Group treated 1 (PRE-MIPS) treated with 1 g·kg^−1^ CHO plus whey protein isolate (0.3 g·kg^−1^) in the form of a recovery shake (mixing with ~200 mL of plain water) within half an hour after completion of exercise. Also, MIPS in the form of a shake (mixing with ~150 mL of plain water) in the 30 min pre-workout (PRE-MIPS, *n* = 10) and ~150 mL the placebo-flavored water (placebo plus flavors only) in the 30 min post-workout.

Group treated 2 (POST-MIPS) treated with 1 g·kg^−1^ CHO plus whey protein isolate (0.3 g·kg^−1^) in the form of a recovery shake (mixing with ~200 mL of plain water) within half an hour after completion of exercise. Also, ~150 mL the placebo-flavored water (placebo plus flavors only) in the 30-min pre-workout and MIPS in the form of a shake (mixing with ~150 mL of plain water) in the in the 30 min’ post- workout (POST-MIPS, *n* = 10).

The proposed doses were chosen based on the safety and efficacy of supplementation in sports medicine [27]. Nutritional supplements are legally classified as food products and are subject to food legislation, in Europe to the EU Directive 2002/46/EC [28] and in the USA to the Dietary Supplement Health and Education Act (DSHEA) [29]. Individuals in each group took the treatments during the 6 days of weekly training in a shake. An independent nutritionist from the study made them with the established composition of individual supplements, so each cyclist and researcher were unaware of which supplements they were consuming. The CHO fraction was comprised of 50% maltodextrin, 25% isomaltose, and 25% fructose (Quamtrax^®^), and the protein source was whey protein (Weider^®^, Weider Global Nutrition Gilbert, 2212 E Williams Field Rd. Ste 230 AZ 85295, USA) mixed with ~200 mL of plain water between 6–12 °C CHO and proteins are commercial products, over-the-counter products. At the same time, the MIPS formulations were made in a pharmacy: creatine monohydrate (5.0 g); L-citrulline malate (6.0 g); L-glutamine (4.0 g); L-taurine (2.0 g); L-arginine (6.0 g); β-alanine (4.0 g); L-ornithine (3 g); L-tyrosine (1.0 g); Bioperine^®^ (black pepper extract (10 mg)). MIPS was mixed with ~150 mL of plain water between 6–12 °C. It was a noncommercial formula that was conceived exclusively for the study. Moreover, all cyclists had already begun supplementing with 10 mg folic acid per day (Interpharma, Barcelona, Spain), 1 g vitamin C per day (Bayer Redoxon^®^, Barcelona, Spain), 1000 µg vitamin B_12_ per day (Solgar S.L., Madrid, Spain), throughout the season. As such, the investigators decided to maintain it during the study intervention; this was part of the supplementation protocol for the *Vuelta a España*.

Although we did not control the hydration status of each cyclist. Hydration recommendations were made by our dietician-nutritionist to ensure optimal hydration status [30]: (i) Pre-exercise: drink 5 to 10 mL·kg^−1^ of body weight in the two to four hours before each exercise session; (ii) During exercise, the hydration goal for most athletes should be customized to avoid a body weight loss of less than 2%; (iii) Rehydration in the next 8 to 24 h post-exercise, athletes should drink 125–150% percent of the total fluid lost during exercise during the six hours immediately following the end of the training session.

### 2.3. Body Composition and Anthropometric Measures

The same internationally certified anthropometrist (ISAK level 3 with certificate number: #63673929292503670742) performed the anthropometric measurements of all cyclists following the protocol of the International Society for the Advancement of Kinanthropometry (ISAK), a worldwide organization based in Glasgow, Scotland (www.isak.global/, accessed on 16 August 2020) [31], at the start of the study. Height (cm) was obtained using a SECA^®^ measuring rod, with an accuracy of 1 mm. Body mass (kg) was measured using a SECA^®^ model scale, accurate to 0.1 kg. Body mass index (BMI) was considered using the equation body mass/height^2^ (kg/m^2^). Six skinfolds (mm) were evaluated: triceps, subscapular, supraspinal, abdominal, anterior thigh, and medial calf, using a Harpenden^®^ skinfold caliper with an accuracy of 0.2 mm, and the sum of all of them was considered. Circumferences (cm) (relaxed arm, flexed arm, minimal waist, thigh 1 cm below the buttock, medial thigh, and calf) were measured with a Lufkin^®^ model W606PM inextensible metal tape measure with an accuracy of 1 mm. Fat mass (FM) and muscle mass (MM) were estimated using the Carter and Lee equations, respectively [31].

### 2.4. Dietary Assessment

Our registered professional dietician-nutritionist carefully recorded the athletes’ daily food and fluid intake throughout the study. All participants were informed about proper food tracking and instructed on two validated methods of dietary recall by the same trained registered dietician-nutritionist. The first method was to complete a food frequency questionnaire (FFQ) at T2, which has been previously utilized for sports populations. The second method was a 7-day dietary recall at T1 and T2 of the 7 days before the test, which was used to examine whether the results of this recall were like that of the FFQ. Food values were then converted into intake of total energy, macronutrients, and micronutrients by a validated software package (Easy diet ©, online version 2019). Likewise, total energy and macronutrients intake in relation to each kg per body mass (BM) was calculated for each athlete [5,16,17,32,33].

### 2.5. Blood Collection and Analysis

All participants attended the laboratory at 8:30 am for blood collection at two specific times, at the baseline of the study (T1) and the end of the study (T2). The World Anti-Doping Agency (WADA) rules for sample collection and transport have been used [34]. Blood samples (10 mL each) were collected from the antecubital vein of all athletes at each point (T1 and T2), under baseline conditions, after an overnight fast and 36 h without exercise. For blood collections, athletes arrived at the laboratory at 8:30 am and upon arrival sat comfortably for 30 min. The blood sample was left at room temperature for 10 min before 15 min of centrifugation at 4 °C and 3000 rpm.

The serum was then separated and stored in aliquots at −20 °C until analysis. All analyses were performed in the same way as other off our previous investigations: CK, LDH, and Mb [5]; Total testosterone (plasma protein-bound testosterone and free testosterone), cortisol and testosterone/cortisol ratio [35]. The percentage changes in plasma volume (% ΔPV) were calculated using Van Beaumont’s equation. In addition, the values of the hematological markers were adjusted for changes in plasma volume [2].

### 2.6. Performance Testing

#### 2.6.1. Aerobic

The test sessions (T1 and T2) were conducted in an indoor sports laboratory under standard conditions (temperature: 21 °C and 60% relative humidity), to maintain the same constants in both tests in order to determine maximal oxygen uptake power (VO2max). We used a Swedish ergometer bike, model Monark 894E, equipped with continuous heart monitoring and a stopwatch. The pedaling frequency was set at 70 rpm. Once the handlebar and saddle were adjusted to each patient’s dimensions, the test began with a 10-min warm-up at a pre-set power of 100 Watts (1.5 Kp). At the end of the warm-up, the heart rate of each subject was measured with a heart-rate monitor (POLAR Ventage M). After the warm-up phase, the subjects completed a 2–5 min recovery phase. During this period, subjects were instructed tto remain seated on the bike. Then the level was increased at a rate of 35 Watt (0.5 Kp) per minute until exhaustion.

Exhaustion was defined as the inability of the athlete to maintain the stipulated speed due to muscular and/or general exhaustion [31]. The initial maximum test load was set at 140 Watt (2 Kp) at a constant speed of 24 km/h per so exhaustion was reached within 5 to 10 min of starting the test. An automated gas analyzer (Vmax 29, Sensormedics, Big Lake, MN, USA) was used to record respiratory parameters every for 20 s, breathing ambient air. The test ended when the ECs could not maintain the pre-set pace of the treadmill. The VO2max (mL kg^−1^·min^−1^) for any 20 s interval was recorded

#### 2.6.2. Anaerobic

The Wingate test [36], was used in a Monark 849E cycloergometer over 30 s to determine the anaerobic threshold. Once the handlebar and saddle were adjusted to the patient’s dimensions, the test began with a 10-min warm-up with a pre-set power of 100 Watts (1.5 Kp). At the end of the warm- up, the subject’s heart rate was measured using a heart rate monitor (POLAR Ventage M). After the warm-up phase, each subject completed a 2 to 5 min recovery phase. During this phase, subjects were instructed to remain seated on the bike and accelerate to maximum speed. Upon the command “GO”, the test began: The Wingate phase consisted of 30 s of maximum cycling intensity at a resistance of 9.0% of the subject’s body weight [36]. Throughout the duration of the Wingate phase, subjects were verbally encouraged to give maximum effort for the full 30 s and were informed of each 5-s interval. Immediately following this interval, resistance was removed, and subjects continued to pedal lightly for 2–3 min.

### 2.7. Statistical Data Analyses

Analyses were performed using STATA version 15.0 (StataCorp, College Station, TX, USA), SPSS software version 24.0 (SPSS, Inc., Chicago, IL, USA), Graphpad Prism (Graphpad Software Version 6.01 San Diego, CA, USA), and Microsoft Excel (Microsoft Excel Software version 19). Data are presented as means and standard deviations. We considered *p*-values of less than 0.05 to be statistically significant. The Shapiro–Wilk test was used to determine normality. Parametric tests were used because the data followed a normal distribution.

The percentage changes of the variables studied in each study group between the T1 and T2 were calculated as Δ (%): ((T2-T1)/T1) × 100) [2]. Barlett and Levene’s tests were applied to measure the equality of the variances. Inter-group comparisons were conducted using one-way analysis of variance (ANOVA). The ANOVA test of repeated two-way measurements was used to examine the effects of interactions (time x supplementation group) between the supplementation groups (CG, PRE-MIPS, and POST-MIPS) for muscle damage (CK, LDH, and Mb) and hormonal responses (testosterone, cortisol, and testosterone/cortisol ratio). The Δ (%) of the study parameters were compared using a unidirectional analysis of covariance with the category of supplements as a fixed factor. A Bonferroni post hoc test was applied for comparisons between groups. Likewise, differences between T1 and T2 in each group were in each group using Student’s *t*-tests for parametric data. Effect sizes among the participants were calculated using a partial eta-square (η2p) [37]. Since this measure is likely to overestimate the effect sizes, the values were interpreted according to indicating no effect if 0 ≤ η2p < 0.05; minimal effect if 0.05 ≤ η2p < 0.26; moderate effect if 0.26 ≤ η2p < 0.64; and strong effect if η2p ≥ 0.64.

## 3. Results

A total of 30 elite male cyclist were included in this analysis. There were no significant differences (*p* > 0.05) between groups in age (years), weight (kg), height (cm), and the sum of six skinfolds (mm) (Table 1).

During the study, the athletes did not show significant statistical differences (*p* > 0.05) in energy, macronutrient, and iron intake among the different groups (Table 2).

Table 3 showed the muscle behavior (CK, LDH and Mb) and hormonal response (testosterone, cortisol, testosterone/cortisol ratio) in the three study groups at the beginning and end of ten weeks of treatment. There was a different behavior of these parameters throughout the study, depending on the treatment group (*p* < 0.05). Also, there were significant differences (*p* < 0.05) between POST-MIPS and CG in T1 in cortisol. Concerning T2, significant differences (*p* < 0.05) observed in all parameters studied between POST-MIPS vs. CG. Moreover, they showed a significant decrease (*p* < 0.05) of CK, LDH, Mb, and cortisol and a significant increase (*p* < 0.05) of testosterone and testosterone/cortisol ratio. A significant decrease between POST-MIPS vs. PRE-MIPS was observed in Mb, cortisol, and testosterone/cortisol ratio, as well as a reduction in CK, LDH. Testosterone and testosterone/cortisol ratios showed an increase in POST-MIPS vs. PRE-MIPS (Table 3).

**Figure 1:**
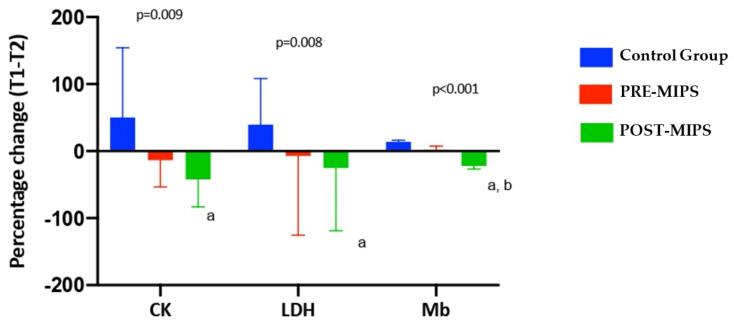
Percentage change in muscle damage parameters between T1-T2. The data are expressed as a mean ± standard deviation. Δ (T1-T2) (%) = ((T2T1)/T1) × 100.CK: Creatine kinase; LDH: Lactate dehydrogenase; Mb: Myoglobin. T1: Before the start of the race; T2: After 10 weeks of treatment. *p*: Group differences obtained through 1-factor ANOVA. ^a^: Significant differences from the Control group (*p* < 0.05). ^b^: Significant differences from the PRE-MIPS group (*p* < 0.05).

Figure 1 shows the percentage change in the parameters of muscle behavior (CK, LDH and Mb) in the three study groups between the baseline and end of treatment (T1-T2). There were significant differences in changes throughout the study between study groups (*p* < 0.05) in all muscle biomarkers. Specifically, there were significant differences in CK POST-MIPS (−42.04 ± 41.05%) vs. CG (50.23 ± 103.83%); in LDH POST-MIPS (−25.03 ± 93.65%) vs. CG (39.54 ± 68.73%); in Mb POST-MIPS (−22.19 ± 4.51%) vs. CG (13.88 ± 2.17%); and POST-MIPS (22.19 ± 4.51%) vs. PRE-MIPS (2.14 ± 5.21%). There was also a greater reduction trend (no significant) in CK POST-MIPS (−42.04 ± 41.05%) vs. PRE-MIPS (−13.36 ± 40.24%) and in LDH POST-MIPS (−25.03 ± 93.65%) vs. CG (39.54 ± 68.73%) (Figure 1).

**Figure 2:**
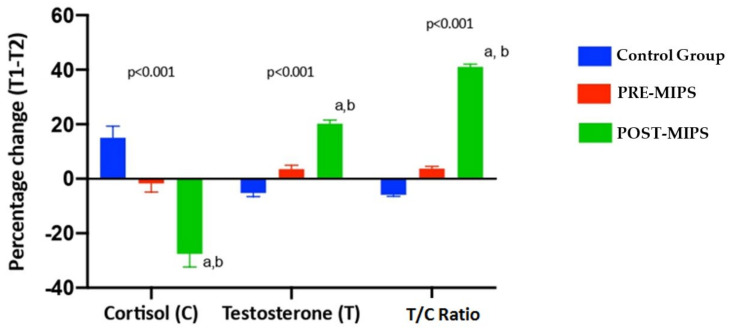
Percentage change in muscle recovery parameters between T1-T2. The data are expressed as a mean ± standard deviation. Δ (T1-T2) (%) = ((T2T1)/T1) × 100. T1: Before the start of the race; T2: After 10 weeks of treatment. *p*: Group differences obtained through a 1-factor ANOVA. ^a^: Significant differences from the Control group (*p* < 0.05). ^b^: Significant differences from the PRE-MIPS (*p* < 0.05).

Figure 2 shows the percentage change in the parameters of hormonal response (testosterone, cortisol, testosterone/cortisol ratio) in the three study groups between the baseline and end of treatment (T1–T2). Figure 2 shows significant differences in percentage change throughout the study between study groups (*p* < 0.05) for hormonal responses. There were significant differences (*p* < 0.05) in cortisol observed POST-MIPS (−27.53 ± 4.83%) vs. CG (15.05 ± 4.27%) and POST-MIPS (−27.53 ± 4.83%) vs. PRE-MIPS (−1.73 ± 3.13%), in testosterone POST-MIPS (20.17 ± 1.38%) vs. CG (−5.17 ± 1.38%) and POST-MIPS (20.17 ± 1.38%) vs. PRE-MIPS (3.54 ± 1.41%), in testosterone/cortisol ratio POST-MIPS (41.08 ± 0.96%) vs. CG (−5.85 ± 0.58%) and POST-MIPS (41.08 ± 0.96%) vs. PRE-MIPS (3.69 ± 0.81%) the (Figure 2).

**Figure 3:**
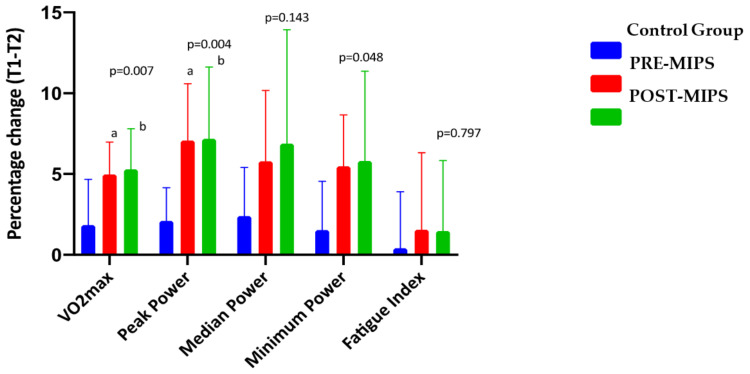
Percentage change in sport performance. The data are expressed as a mean ± standard deviation. Δ (T1-T2) (%) = ((T2T1)/T1) × 100. T1: Before the start of the race; T2: After 10 weeks of treatment. *p*: *p*-value between groups. Significant values (*p* < 0.005). ^a^: Significant differences from the Control group (*p* < 0.05). ^b^: Significant differences from the PRE-MIPS (*p* < 0.05).

Figure 3 shows the percentage change in performance parameters between the beginning and end of the follow-up of the study. Significant differences were observed in VO_2_max (ml/kg(min)) (*p* = 0.007), peak power (W) (*p* = 0.004), and minimum power (W) (*p* = 0.048) between groups. These data show significant differences (*p* < 0.05) in VO_2_max POST-MIPS (5.30 ± 2.51%) vs. CG (1.84 ± 2.84%) and PRE-MIPS (4.98 ± 2.00%) vs. CG (1.84 ± 2.84%); in Peak Power POST-MIPS (7.18 ± 4.44%) vs. CG (2.10 ± 2.05%) and PRE-MIPS (7.08 ± 3.52%) vs. CG (2.10 ± 2.05%). The results did not show significant differences between groups in the remainder of performance markers (Media Power, Minimum Power, and Fatigue Index).

## 4. Discussion

This is the first study to our knowledge that describes the effects of MIPS supplementation associated with CHOs plus whey protein isolate (post-workout recommendations) [9,10] on muscle behavior, hormonal response, and sports performance in elite cyclists. Moreover, the impact of the optimal timing of MIPS’ supplementation was investigated. The amounts of the active ingredients follow the regular dosage and safety recommendations of all active ingredients like creatine monohydrate, L- citrulline malate, L-glutamine, L-taurine, L-arginine, β-alanine, L-ornithine, L-tyrosine [8,13,14,25,27,38,39], and bioavailability enhancers such as black pepper extract [13,40]. In MIPS formed by a set of ingredients in a single formulation, it is necessary that all components of MIPS are included in effective and safe doses because it is determinant in the potential effect in the study [13].

Researchers are responsible for guaranteeing the safety of MIPS. It is not obligatory, however, to provide data that demonstrate safety and efficacy to the European Food Safety Agency (EFSA) [28], Federal Drug Administration (FDA) [41], and/or the Spanish Agency for Medicines and Health Products (AEMPS) [27]. In our study, no side effects from supplementation, in each group, were reported. No ingredients used were WADA-prohibited substances [12].

Intense and prolonged cycling induces exercise-induced muscle damage (EIMD), favoring the release of muscle-damaging proteins such as serum CK, LDH, and Mb into the bloodstream [42], as well as hormones such as cortisol, which are involved in the activation of catabolic processes and anti-anabolic actions related to protein turnover [43]. These biomarkers can provide information regarding the athletes’ physiological responses [44] and the effectiveness of nutritional-recovery strategies [35]. In all cyclists in the study, CK values before training camp are already above the clinical reference range. The cyclists in the study could present elevated levels of CK due to the continuous concentric muscle contraction in extensive aerobic conditions developed during the competition season. Such cases of elevated levels of markers of muscle damage are common in elite athletes and especially toward the end of the season because of several consecutive months of demanding physical activity [45]. For cyclists in our study, the supplementation with CHO plus proteins was not sufficient to attenuate EIMD (CK, LDH and Mb were significantly increased). Nor was it sufficient to modulate the catabolic response by significantly increasing cortisol and significantly decreasing testosterone and testosterone/cortisol ratio between T1 and T2. This could be because, in strenuous exercise situations, CHO plus proteins supplementation is insufficient and additional supplementation is necessary [12]. The protein intake of the cyclists was 2.3–2.5/kg body weight, and the CHO intake was 8.2–8.5/kg body weight. Both intakes exceed the established recommendations to stimulate metabolic adaptation, tissue repair, protein turnover, and increase the rate of muscle glycogen resynthesis [6,7]. Therefore, we believe that supplementation with MIPS is necessary and even increases the value of the recommendations for this type of extreme physical exertion. Thus, the results of our study on elite cyclists indicate that intake of MIPS pre- and post-workout was influential in attenuating muscle damage, stimulating anabolic hormone response and modulating catabolic hormone response. We also found that the ingestion of MIPS post-workout (POST-MIPS) was more effective in improving muscle maintenance and hormonal response than PRE-MIPS. These data suggest showed that our MIPS could delay fatigue, improve recovery, and preserve muscle integrity when supplemented after training. This could be explained by the fact that the leucine trigger hypothesis, from whey protein isolate, is insufficient for muscle protein synthesis (MPS) in our study. The leucine trigger hypothesis is because protein administration, especially whey protein isolate, increases blood concentrations of essential amino acids, mainly leucine, and promotes MPS mediated by activation of the target complex of rapamycin 1 (mTORC1) [46,47]. However, there are some considerations that would modify leucine concentration (the dose and source of protein; the type and intensity of exercise), derived from protein supplementation, which could alter MPS [46]. Therefore, the differences observed in the groups supplemented with MIPS compared to GC would justify supplementation with MIPS, because MPS would be increased attenuating muscle damage. MPS could be additive (MIPS contain BCAAs) or synergistic by the combination of other MIPS ingredients.

With respect the ergogenic effects of MIPS consumption on sports performance, significant differences were observed in the percentage of change in performance parameters (VO2max, peak power, and minimum power) between groups. These results are in line with other MIPS studies observing positive effects on aerobic and anaerobic sports performance and fatigue assessment [13,48,49,50,51]. Daily ingestion for ten weeks of a pre-workout (PRE-MIPS) and post-workout supplement (POST-MIPS) blend showed positive effects on sports performance, more than a CHO plus proteins (CG), when combined with ten weeks cycling training sessions. Improvements in VO2max and peak power were significant (<0.05) in PRE-MIPS and POST-MIPS with respect to CG, median power, minimum power, and fatigue index there were a trend of improvement only in the groups supplemented with MIPS (PRE-MIPS and POST-MIPS). In our study, PRE-MIPS and POST-MIPS did not show significant differences in exercise performance. Nonetheless, the performance outcomes were modest in POST-MIPS compared to PRE-MIPS in elite cyclists. Sports performance improvements were probably due to reduced muscle damage [52,53] and stimulation of anabolic hormone [51] action attributable to the MIPS’ content. MIPS supplementation appears to be effective in increasing the energy contribution on aerobic and anaerobic metabolism and increasing energy expenditure would increase the time to exhaustion [54]. In addition, ingestion of ingested MIPS would allow cyclists to endure higher plasma lactate levels and for longer periods of time. This would delay the onset of fatigue. Perhaps, these effects are due to L-citrulline, which could improve performance and increase muscular endurance due to increased ammonium clearance and reduced lactate accumulation [48,49,55].

The reason for not showing significant differences between the two supplemented groups in our study in cyclists could be because supplementation pre-training has shown improvements in mainly anaerobic exercise [27], and supplementation post-training has benefited endurance exercise [14,56]. Our cyclists practice a multimodal sport due to the demands of the varied routes and, therefore, use aerobic metabolism on the flat or anaerobic metabolism when sprinting and climbing mountains [18]. Another hypothesis, as to why no significant differences were observed between the PRE-MIPS and POST-MIPS groups, could be due to the fact that ten weeks of supplementation may be sufficient to obtain adequate plasma concentrations of MIPS ingredients, regardless of the timing of intake [27].

Other studies that used MIPS formulas containing similar mixes of ingredients have demonstrated positive results in sports performance [57,58,59,60], reduction in EIMD biomarkers [60,61], modulation of hormonal response [14,62], and recovery [57,60]. These findings agree with ours. Such results have been found in different sports such as running, taekwondo, tennis, swimming [14,57,59,60,61,62]. Alternatively, one study [8] reported that MIPS consumption for four weeks had no effect on damage muscle in endurance-trained males or females. Moreover, regular MIPS supplementation has shown no effect in resistance-trained males, on increase testosterone or decrease in cortisol concentration relative to placebo [8]. Therefore, caution should be exercised when extrapolating these results because the specificity of the effort must be considered in relation to the muscular component of the contraction (concentric or eccentric) [63], taking into account that cyclists perform a continuous concentric muscular contraction [5,32].

In MIPS, there are several ingredients that are found in the formulations of numerous preparations, but not all MIPS contain the same compounds [8]. Although there are certain common ingredients, the addition of complementary products can modify the mechanisms of action of MIPS and the potential muscular, hormonal, body composition, and sports performance benefits [39]. Also, MIPS administered to types of athletes with different physiological demands are quite different and are associated with specific sports in which one or another component of physical activity is used (aerobic or anaerobic) [64]. Another aspect of MIPS research is that administration time also differs [19,20]. For these reasons, they complicate efforts to benchmark MIPS. However, the ergogenic effects of each MIPS will depend on the specific of ingredients in its formulation.

β-alanine is a non-essential amino acid that alone has no ergogenic effect. Nonetheless, it significantly increases carnosine concentrations in skeletal muscle. Improvement of exercise performance, at doses of 4–6 g/day for at least 2–4 weeks [38] potentially improves muscle contraction, increases the sensitivity of myofibrillary calcium in fast fibers (IIa and IIb), and acts as a buffer of the intracellular pH against the accumulation of protons in the muscle, reducing the performance-limiting effect related to acidosis in continuous or intermittent activities of between 30 s and 10 min [8,64]. Eight-week β-Alanine supplementation improves cyclists’ performance by improving the maximum power in the sprints used at the end of the races [65]. As in our study, which significantly increased the Wingate test. Other studies reported a synergistic effect when combining β-alanine plus creatine on improved recovery capacity, delaying fatigue, increased cardiorespiratory endurance improvements, and, overall, improved the stimulus for training [38,64,66,67]. Monotherapeutic creatine supplementation provides a safe and effective nutritional strategy for increasing sports performance (Performance Supplement Group A) [6]. This substances is one of the most widely used supplements by athletes and has a 50% prevalence in MIPS formulations [39]. Creatine improves recovery by stimulating muscle protein synthesis, increasing testosterone levels, reducing post-training lactate concentration, and modulating CK, Mb, and LDH (muscle damage markers) in highly trained athletes [16,17,68], findings that are similar to our results.

Nitric oxide (NO) production is enhanced by some of the components most used by MIPS, such as L-Arginine and L-Citrulline. It stimulates vasodilation by improving blood flow, is involved in skeletal-muscle energy metabolism via stimulating mitochondrial oxidation, and has restorative properties in EIMD [13,69]. Such properties attributed to NO could be responsible for increased sports performance [70]. Supplementation with L-Citrulline has a dual effect by simultaneously increasing plasma levels of L-Arginine and L-Citrulline [71]. Alternatively, arginine is attributed to a growth hormone (GH) and insulin stimulating effect, while acting as a creatine precursor [72,73]. Furthermore, combined supplementation of L-Arginine plus L-Ornithine in weight lifters and body builders (strength-athletes) for 21 days stimulated (1-h post-exercise) significant increases in GH and insulin growth factor 1 (IGF-1) [74]. Another potential synergism is through the simultaneous supplementation of three ingredients, such as L-Citrulline, L-Arginine, and L-Ornithine, which have shown a decrease in plasma ammonia values, which increases the tolerance of the organism to intense exercise and accelerate recovery processes [38,75]. Moreover, L-Citrulline, L-Arginine, and L-Ornithine increase muscular glycogen and glutamine synthesis while decreasing post-exercise blood lactate levels and improving overall sport performance [62,76].

L-Glutamine is the most abundant amino acid in skeletal muscles and plasma, constituting approximately 60% of the total free amino acids in the former skeletal and 20% in the latter. L-Glutamine might play a role in the synthesis of other amino acids (alanine and aspartate), proteins, and a number of other biological molecules such as nucleotide (purines, pyrimidines, and amino sugars), nicotinamide adenine dinucleotide phosphate (NADPH), glutathione, and antioxidants [77]. L-Glutamine also appears to have anti-inflammatory properties, a feature associated with a reduction of indirect markers EIMD, a reduction of delayed onset muscle soreness (DOMS), and improved muscular function [78]. It has a synergistic effect on various MIPS by fatigue delay and accelerated elimination of fatigue-related metabolites [77,79]. Chen et al. [79], for example, found enhanced serum total protein (TP) content with MIPS supplementation with only L-Arginine and L-Glutamine stimulated muscle protein synthesis. In this study [79], lactate, ammonia, glucose, and CK levels were positively modulated by MIPS supplementation and were dose-dependent for ammonia, glucose, and CK.

L-Tyrosine supplementation was associated with lower Borg Rating of Perceived Exertion (RPE) scores, likely due to higher levels of motivation in athletes as a consequence of optimal neurotransmitter levels [80]. L-Taurine has antioxidant effects, which allows the reduction of biomarkers of muscle damage and DOMS. Regular L-Taurine supplementation improves time to exhaustion, and one-time ingestion improves muscular efficiency in endurance athletes [8]. One ingredient of our MIPS was Bioperine^®^, which contains piperine (1-(1-(1,3-benzodioxol-5-yl)-1-oxo-2,4 pentenyl piperidine), an active principle obtained from black pepper. Piperine acts as a thermonutrient that allows for increased absorption and bioavailability of the MIPS ingredients [40].

The limitation of our study lies in the use of a mixture (creatine monohydrate, L-citrulline malate, L-glutamine, L-taurine, L-arginine, β-alanine, L-ornithine, L-tyrosine) that complicates understanding how each component contributes to improving the study results and exerting a potential synergistic effect, although the potential impacts of different ingredients in monotherapy have been previously reported [27]. MIPS may also produce response variations due to interindividual variability or MIPS exerts no detectable effect. In this sense, only creatine and β-alanine may have a positive effect on the athlete’s body and performance [81]. These results are when the MIPS ingredients have been evaluated in monotherapy. However, we reported that double combinations of β-hydroxy-β-methylbutyrate (HMB) + creatine have shown a synergistic effect in improving athletic performance and reducing cortisol [16,17]. These MIPS supplementation studies could enhance the effects on biomarkers and/or sports performance by a synergistic effect, which would result in the activation of several pathways simultaneously that would improve the effect of single-ingredient supplementation. Although they are different fields, the pharmacological combination is frequently used in different fields of medicine, as in medical oncology, with the use of immunomodulatory agents (IMIDs) plus dexamethasone [82,83]. Also, because the hydration status of each cyclist was not controlled, some hydration recommendations were established to avoid deterioration of sports performance. Body mass was not measured after the study.

In conclusion, our MIPS supplementation could offer a practical and convenient way to improve recovery and athletic performance in elite cyclists who compete and/or train on consecutive days. Nonetheless, more longitudinal, and experimental studies with longer follow-up are needed to unravel the association between MIPS on exercise-induced fatigue-related parameters and performance improvements.

### Practical Applications

This research could be of interest to sport physicians, nutritionists, and coaches who want to improve sports performance, muscle recovery after exercise, and the hormonal status of their athletes. Considering the described optimal timing, the composition of our MIPS: Creatine monohydrate (5.0 g); L-Citrulline malate (6.0 g); L-Glutamine (4.0 g); L-Taurine (2.0 g); L-Arginine (6.0 g); β-Alanine (4.0 g); L-Ornithine (3 g); L-Tyrosine (1.0 g); Bioperine^®^ (black pepper extract (10 mg); and their combined long-term effects, supplementation for ten weeks, as a strategy in critical phases of supplementation during periods of intense and strenuous training. Most high-performance athletes ingest different supplements simultaneously at different times and doses. Multi-ingredient formulations such as the one presented in this study may provide a convenient way for athletes in particular settings where logistics may be more complicated. These encouraging results represent an important improvement in supplementation strategies intended to improve markers of muscle damage, hormonal responses, and athletic performance, while improving adherence, cost-reduction, and minimizing errors in dosage. The dose and duration of supplementation should always reflect individual requirement, and it should be always monitored by a dietitian and/or nutritionist.

## Figures and Tables

**Table 1:** Anthropometry and body composition data in the three study groups at baseline of study.

	Control Group (CG)	PRE-MIPS Group	POST-MIPS Group	*p*
Sample size (*n*)	10	10	10	
Age (years)	26.1 ± 4.6	25.7 ± 6.4	27.7 ± 2.4	0.801
Weight (kg)	66.3 ± 4.6	65.9 ± 3.9	64.9 ± 4.7	0.833
Height (cm)	176.1 ± 3.8	174.2 ± 4.3	172.2 ± 6.3	0.881
Σ6 skinfolds (mm)	31.2 ± 4.5	32.4 ± 6.2	33.4 ± 7.2	0.911

Data are expressed as mean ± standard deviation. *p*: Differences between groups using one-way ANOVA.

**Table 2:** Energy, macronutrient, and iron intake in the three study groups during 10 weeks of study.

	Control Group (CG)	PRE-MIPS Group	POST-MIPS Group	*p*
Sample size (*n*)	10	10	10	
Energy (kcal)	3175 ± 395	3190 ± 410	3268 ± 358	0.693
Energy (kcal/kg)	43.1 ± 7.0	42.7 ± 6.3	41.0 ± 6.6	0.126
Protein (g)	152.8 ± 25.6	150.5 ± 29.4	154.6 ± 25.5	0.786
Protein (%)	16.2 ± 2.9	17.0 ± 3.2	16.9 ± 2.7	0.318
Protein (g/kg)	1.7 ± 0.4	1.7 ± 0.7	1.8 ± 0.6	0.830
Animal protein (g)	83.0 ± 25.3	83.1 ± 19.3	82.6 ± 24.6	0.531
Vegetal protein (g)	57.4 ± 16.5	60.3 ± 19.1	59.3 ± 15.3	0.494
Fat (g)	91.3 ± 21.8	93.8 ± 20.8	92.6 ± 21.0	0.252
Fat (%)	26.5 ± 5.1	26.2 ± 4.8	25.6 ± 4.2	0.269
Fat (g/kg)	1.3 ± 0.6	1.4 ± 0.6	1.2 ± 0.5	0.254
Total carbohydrates (g)	552.9 ± 60.5	558.5 ± 58.2	560.2 ± 60.1	0.745
Carbohydrates (%)	64.2 ± 4.7	64.8 ± 6.1	65.0 ± 5.2	0.720
Carbohydrates (g/kg)	7.0 ± 1.2	7.1 ± 1.3	7.1 ± 1.1	0.980
Iron (Fe) (mg)	33.3 ± 7.5	34.0 ± 6.9	33.9 ± 7.1	0.611

Data are expressed as mean ± standard deviation. *p*: Differences between groups using one-way ANOVA.

**Table 3:** Behavior of muscle damage and muscle recovery in the 3 study groups at the beginning and end of 10 weeks of treatment.

	T1	T2	*p* (TXG)	η2p
	CREATINE KINASE (U/L)			
CG *	225.82 ± 117.82	336.83 ± 302.70	0.005	0.191
PRE-MIPS	274.56 ± 267.12	238.89 ± 159.86		
POST-MIPS *	275.89 ± 189.28	163.89 ± 103.18 ^a^		
	**LACTATE DEHYDROGENASE (U/L)**			
CG *	391.19 ± 72.49	409.77 ± 73.90	0.008	0.201
PRE-MIPS	342.43 ± 110.15	318.43 ± 100.53		
POST-MIPS *	357.68 ± 113.59	271.1212 ± 98.76 ^a^		
	**MYOGLOBIN (ng·mL^−1^)**			
CG *	21.60 ± 1.78	24.60 ± 2.08	<0.001	0.197
PRE-MIPS	24.43 ± 5.40	24.93 ± 5.10		
POST-MIPS *	25.43 ± 4.30	19.77 ± 4.74 ^a,b^		
	**CORTISOL (µg·dL^−1^)**			
CG *	18.16 ± 3.04	20.71 ± 3.96	<0.001	0.329
PRE-MIPS	19.86 ± 2.83	19.52 ± 2.99		
POST-MIPS *	21.31 ± 5.03 ^a^	15.67 ± 2.66 ^a,b^		
	**TOTAL TESTOSTERONE (ng·dL^−1^)**			
CG *	6.18 ± 1.33	5.87 ± 1.80	<0.001	0.282
PRE-MIPS	5.22 ± 1.37	5.41 ± 1.71		
POST-MIPS *	4.71 ± 1.04	5.66 ± 1.13 ^a^		
	**TESTOSTERONE/CORTISOL RATIO**			
CG *	0.34 ± 0.33	0.32 ± 0.88	<0.001	0.300
PRE-MIPS	0.27 ± 0.39	0.26 ± 0.76		
POST-MIPS *	0.22 ± 0.875	0.31 ± 0.62 ^a,b^		

The data are expressed as a mean ± standard deviation. CG: Control Group. T1: Baseline; T2: After ten weeks of treatment. *p*: Interaction time per group obtained by means of 2-factor ANOVA. η2p: The effect of the size. *: Significant differences between study phases (T1 vs. T2) (*p* < 0.05). ^a^: Significant differences from the CG group (*p* < 0.05). ^b^: Significant differences from the PRE-MIPS group (*p* < 0.05).

## References

[1] Jeukendrup A.E., Craig N.P., Hawley J.A. (2000). The bioenergetics of world class cycling. J. Sci. Med. Sport.

[2] Fernández-Lázaro D. (2020). Ergogenic Strategies for Optimizing Performance and Health in Regular Physical Activity Participants: Evaluation of the Efficacy of Compressive Cryotherapy, Exposure to Intermittent Hypoxia at Rest and Sectorized Training of the Inspiratory Muscles. Ph.D. Thesis.

[3] Saw A., Halson S., Mujika I. (2018). Monitoring athletes during training camps: Observations and translatable strategies from elite road cyclists and swimmers. Sports.

[4] Hansen M., Bangsbo J., Jensen J., Krause-Jensen M., Bibby B.M., Sollie O., Hall U.A., Madsen K. (2016). Protein intake during training sessions has no effect on performance and recovery during a strenuous training camp for elite cyclists. J. Int. Soc. Sports Nutr..

[5] Córdova A., Mielgo-Ayuso J., Fernandez-Lazaro C.I., Caballero-García A., Roche E., Fernández-Lázaro D. (2019). Effect of iron supplementation on the modulation of iron metabolism, muscle damage biomarkers and cortisol in professional cyclists. Nutrients.

[6] Thomas D.T., Erdman K.A., Burke L.M. (2016). Position of the Academy of Nutrition and Dietetics, Dietitians of Canada, and the American College of Sports Medicine: Nutrition and athletic performance. J. Acad. Nutr. Diet..

[7] Meeusen R., Duclos M., Foster C., Fry A., Gleeson M., Nieman D., Raglin J., Rietjens G., Steinacker J., Urhausen A. (2013). Prevention, diagnosis and treatment of the overtraining syndrome: Joint consensus statement of the European College of Sport Science (ECSS) and the American College of Sports Medicine (ACSM). Eur. J. Sport Sci..

[8] Harty P.S., Zabriskie H.A., Erickson J.L., Molling P.E., Kerksick C.M., Jagim A.R. (2018). Multi-ingredient pre-workout supplements, safety implications, and performance outcomes: A brief review. J. Int. Soc. Sports Nutr..

[9] Calleja-González J., Mielgo-Ayuso J., Sampaio J., Delextrat A., Ostojic S.M., Marques-Jiménez D., Arratibel I., Sánchez-Ureña B., Dupont G., Schelling X. (2018). Brief ideas about evidence-based recovery in team sports. J. Exerc. Rehabil..

[10] Murray B., Rosenbloom C. (2018). Fundamentals of glycogen metabolism for coaches and athletes. Nutr. Rev..

[11] Vitale K., Getzin A. (2019). Nutrition and supplement update for the endurance athlete: Review and recommendations. Nutrients.

[12] Moriones V.S., Santos J.I. (2017). Ergogenic aids in sport. Nutr. Hosp..

[13] Kaczka P., Batra A., Kubicka K., Maciejczyk M., Rzeszutko-Bełzowska A., Pezdan-Śliż I., Michałowska-Sawczyn M., Przydział M., Płonka A., Cięszczyk P. (2020). Effects of p6e-workout multi-ingredient supplement on anaerobic performance: Randomized double-blind crossover study. Int. J. Environ. Res. Public Health.

[14] Ormsbee M.J., Mandler W.K., Thomas D.D., Ward E.G., Kinsey A.W., Simonavice E., Panton L.B., Kim J.S. (2012). The effects of six weeks of supplementation with multi-ingredient performance supplements and resistance training on anabolic hormones, body composition, strength, and power in resistance-trained men. J. Int. Soc. Sports Nutr..

[15] Directo D., Wong M.W.H., Elam M.L., Falcone P., Osmond A., Jo E. (2019). The effects of a multi-ingredient performance supplement combined with resistance training on exercise volume, muscular strength, and body composition. Sports.

[16] Fernández-Landa J., Fernández-Lázaro D., Calleja-González J., Caballero-García A., Córdova A., León-Guereño P., Mielgo-Ayuso J. (2020). Long-term effect of combination of creatine monohydrate plus β-hydroxy β-methylbutyrate (HMB) on exercise-induced muscle damage and Anabolic/Catabolic hormones in elite male endurance athletes. Biomolecules.

[17] Fernández-Landa J., Fernández-Lázaro D., Calleja-González J., Caballero-García A., Córdova Martínez A., León-Guereño P., Mielgo-Ayuso J. (2020). Effect of ten weeks of creatine monohydrate plus HMB supplementation on athletic performance tests in elite male endurance athletes. Nutrients.

[18] Lucía A., Hoyos J., Chicharro J.L. (2001). Physiology of professional road cycling. Sports Med..

[19] Arent S.M., Cintineo H.P., McFadden B.A., Chandler A.J., Arent M.A. (2020). Nutrient timing: A garage door of opportunity?. Nutrients.

[20] Ivy J.L., Ferguson-Stegall L.M. (2014). Nutrient timing: The means to improved exercise performance, recovery, and training adaptation. Am. J. Lifestyle Med..

[21] Kerksick C., Harvey T., Stout J., Campbell B., Wilborn C., Kreider R., Kalman D., Ziegenfuss T., Lopez H., Landis J. (2008). International Society of Sports Nutrition position stand: Nutrient timing. J. Int. Soc. Sports Nutr..

[22] Lemon P.W.R., Berardi J.M., Noreen E.E. (2002). The role of protein and amino acid supplements in the athlete’s diet: Does type or timing of ingestion matter?. Curr. Sports Med. Rep..

[23] Munteanu A.M., Manuc D., Caramoci A., Vasilescu M., Ionescu A. (2014). Nutrition timing in top athletes. Sport Med. J..

[24] Spillane M., Schwarz N., Leddy S., Correa T., Minter M., Longoria V., Willoughby D.S. (2011). Effects of 28 days of resistance exercise while consuming commercially available pre-and post-workout supplements, NO-Shotgun^®^ and NO-Synthesize^®^ on body composition, muscle strength and mass, markers of protein synthesis, and clinical safety markers in males. Nutr. Metab..

[25] Ormsbee M.J., Thomas D.D., Mandler W.K., Ward E.G., Kinsey A.W., Panton L.B., Scheett T.P., Hooshmand S., Simonavice E., Kim J.S. (2013). The effects of pre-and post-exercise consumption of multi-ingredient performance supplements on cardiovascular health and body fat in trained men after six weeks of resistance training: A stratified, randomized, double-blind study. Nutr. Metab..

[26] Mielgo-Ayuso J., Maroto-Sánchez B., Luzardo-Socorro R., Palacios G., Palacios N., González-Gross M., EXERNET Study Group (2015). Evaluation of nutritional status and energy expenditure in athletes. Rev. Esp. Nutr. Comunitaria.

[27] Palacios Gil de Antuñano N., Manonelles Marqueta P., Blasco Redondo R., Contreras Fernández C., Franco Bonafonte L., Gaztañaga Aurrekoetxea T., Manuz González B., Teresa Galván C., del Valle Soto M. (2019). Nutritional Supplements for the Athlete. Ergogenic aids in sport-2019. Consensus document of the Spanish Society of Sports Medicine. Arch. Med. Deporte.

[28] European Food Safety Authority Nutrition Applications: Regulations and Guidance. https://www.efsa.europa.eu/en/applications/nutrition/regulationsandguidance.

[29] U.S. Food and Drug Administration (1994). Dietary Supplement Health and Education Act of 1994. Technical Report. https://ods.od.nih.gov/About/DSHEA_Wording.aspx.

[30] Sawka M.N., Burke L.M., Eichner E.R., Maughan R.J., Montain S.J., Stachenfeld N.S. (2007). American College of sports medicine position stand: Exercise and fluid replacement. Med. Sci. Sports Exerc..

[31] Stewart A., Marfell-Jones M., Olds T., Ridder H. (2011). International standards for anthropometric assessment. Int. Soc. Adv. Kinanthropometry.

[32] Córdova A., Mielgo-Ayuso J., Roche E., Caballero-García A., Fernandez-Lázaro D. (2019). Impact of magnesium supplementation in muscle damage of professional cyclists competing in a stage race. Nutrients.

[33] Fernández-Lázaro D., Mielgo-Ayuso J., Caballero-García A., Martínez A.C., Seco-Calvo J., Fernández-Lázaro C.I. (2020). Compressive cryotherapy as a non-pharmacological muscle recovery strategy with no adverse effects in basketball. Arch. Med. Deporte.

[34] World Anti-Doping Agency (2016). Guidelines—Blood Sample Collection. https://www.wada-ama.org/en/resources/world-anti-doping-program/guidelines-blood-sample-collection.

[35] Córdova A., Fernández-Lázaro D., Mielgo-Ayuso J., Seco-Calvo J., Caballero-García A. (2017). Effect of magnesium supplementation on muscular damage markers in basketball players during a full season. J. Magnes. Res..

[36] Kenney W.L., Wilmore J.H., Costill D.L. (2008). Physiology of Sport and Exercise.

[37] Ferguson C.J. (2016). An effect size primer: A guide for clinicians and researchers. Prof. Psychol. Res. Pract..

[38] Eudy A.E., Gordon L.L., Hockaday B.C., Lee D.A., Lee V., Luu D., Martinez C.A., Ambrose P.J. (2013). Efficacy and safety of ingredients found in preworkout supplements. Am. J. Heal. Pharm..

[39] Jagim A.R., Harty P.S., Camic C.L. (2019). Common ingredient profiles of multi-ingredient pre-workout supplements. Nutrients.

[40] Fernández-Lázaro D., Mielgo-Ayuso J., Córodova A., Seco-Calvo J. (2020). Iron and physical activity: Bioavailability enhancers, properties of black pepper (bioperine^®^) and potential applications. Nutrients.

[41] Food and Drug Administration (FDA) Information for Consumers on Using Dietary Supplements|FDA. https://www.fda.gov/food/dietary-supplements/information-consumers-using-dietary-supplements.

[42] Fernández-Lázaro D., Mielgo-Ayuso J., Calvo J.S., Martínez A.C., García A.C., Fernandez-Lazaro C.I. (2020). Modulation of exercise-induced muscle damage, inflammation, and oxidative markers by curcumin supplementation in a physically active population: A systematic review. Nutrients.

[43] Fernández-Lázaro D., Fernandez-Lazaro C.I., Mielgo-Ayuso J., Navascués L.J., Martínez A.C., Seco-Calvo J. (2020). The role of selenium mineral trace element in exercise: Antioxidant defense system, muscle performance, hormone response, and athletic performance. A systematic review. Nutrients.

[44] Fernández-Lázaro D., González-Bernal J.J., Sánchez-Serrano N., Navascués L.J., Ascaso-del-Río A., Mielgo-Ayuso J. (2020). Physical exercise as a multimodal tool for COVID-19: Could it be used as a preventive strategy?. Int. J. Environ. Res. Public Health.

[45] Fernández-Lázaro D., Gallego-Gallego D., Corchete L.A., Fernández Zoppino D., González-Bernal J.J., García Gómez B., Mielgo-Ayuso J. (2021). Inspiratory muscle training program using the powerbreath^®^: Does it have ergogenic potential for respiratory and/or athletic performance? a systematic review with meta-analysis. Int. J. Environ. Res. Public Health.

[46] Zaromskyte G., Prokopidis K., Ioannidis T., Tipton K.D., Witard O.C. (2021). Evaluating the leucine trigger hypothesis to explain the post-prandial regulation of muscle protein synthesis in young and older adults: A systematic review. Front. Nutr..

[47] Suryawan A., Jeyapalan A.S., Orellana R.A., Wilson F.A., Nguyen H.V., Davis T.A. (2008). Leucine stimulates protein synthesis in skeletal muscle of neonatal pigs by enhancing mTORC1 activation. Am. J. Physiol. Endocrinol. Metab..

[48] Fye H., Pass C., Dickman K., Bredahl E., Eckerson J., Siedlik J. (2021). The effect of a multi-ingredient pre-workout supplement on time to fatigue in NCAA division I cross-country athletes. Nutrients.

[49] Pasiakos S.M., McLellan T.M., Lieberman H.R. (2015). The effects of protein supplements on muscle mass, strength, and aerobic and anaerobic power in healthy adults: A systematic review. Sports Med..

[50] Jagim A.R., Jones M.T., Wright G.A., Antoine C.S., Kovacs A., Oliver J.M. (2016). The acute effects of multi-ingredient pre-workout ingestion on strength performance, lower body power, and anaerobic capacity. J. Int. Soc. Sports Nutr..

[51] Schwarz N.A., McKinley-Barnard S.K. (2020). Acute oral ingestion of a multi-ingredient preworkout supplement increases exercise performance and alters Postexercise hormone responses: A randomized crossover, double-blinded, placebo-controlled trial. J. Diet. Suppl..

[52] Naclerio F., Larumbe-Zabala E., Cooper R., Jimenez A., Goss-Sampson M. (2014). Effect of a carbohydrate-protein multi-ingredient supplement on intermittent sprint performance and muscle damage in recreational athletes. Appl. Physiol. Nutr. Metab..

[53] Naclerio F., Larumbe-Zabala E., Cooper R., Allgrove J., Earnest C.P. (2015). A multi-ingredient containing carbohydrate, proteins L-glutamine and L-carnitine attenuates fatigue perception with no effect on performance, muscle damage or immunity in soccer players. PLoS ONE.

[54] Figueiredo C., Lira F.S., Rossi F.E., Billaut F., Loschi R., Padilha C.S. (2020). Multi-ingredient pre-workout supplementation changes energy system contribution and improves performance during high-intensity intermittent exercise in physically active individuals: A double-blind and placebo controlled study. J. Int. Soc. Sports Nutr..

[55] Rhim H.C., Kim S.J., Park J., Jang K.M. (2020). Effect of citrulline on post-exercise rating of perceived exertion, muscle soreness, and blood lactate levels: A systematic review and meta-analysis. J. Sport Health Sci..

[56] Naclerio F., Seijo M., Earnest C.P., Puente-Fernández J., Larumbe-Zabala E. (2020). Ingesting a Post-Workout Vegan-Protein Multi-Ingredient Expedites Recovery after Resistance Training in Trained Young Males. J. Diet. Suppl..

[57] Cheng I.-S., Wang Y.-W., Chen I.-F., Hsu G.-S., Hsueh C.-F., Chang C.-K. (2016). The supplementation of branched-chain amino acids, arginine, and citrulline improves endurance exercise performance in two consecutive days. J. Sports Sci. Med..

[58] Gervasi M., Sisti D., Amatori S., Zeppa S.D., Annibalini G., Piccoli G., Vallorani L., Benelli P., Rocchi M.B.L., Barbieri E. (2020). Effects of a commercially available branched-chain amino acid-alanine-carbohydrate-based sports supplement on perceived exertion and performance in high intensity endurance cycling tests. J. Int. Soc. Sports Nutr..

[59] Hsueh C.-F., Wu H.-J., Tsai T.-S., Wu C.-L., Chang C.-K. (2018). The effect of branched-chain amino acids, citrulline, and arginine on high-intensity interval performance in young swimmers. Nutrients.

[60] Yang C., Wu C., Chen I., Chang C. (2017). Prevention of perceptual-motor decline by branched-chain amino acids, arginine, citrulline after tennis match. Scand. J. Med. Sci. Sports.

[61] Chen I.-F., Wu H.-J., Chen C.-Y., Chou K.-M., Chang C.-K. (2016). Branched-chain amino acids, arginine, citrulline alleviate central fatigue after 3 simulated matches in taekwondo athletes: A randomized controlled trial. J. Int. Soc. Sports Nutr..

[62] Puente-Fernández J., Seijo M., Larumbe-Zabala E., Jiménez A., Liguori G., Rossato C.J.L., Mayo X., Naclerio F. (2020). Effects of multi-ingredient preworkout supplementation across a five-day resistance and endurance training microcycle in middle-aged adults. Nutrients.

[63] Fernández-Lázaro D., Mielgo-Ayuso J., Adams D.P., González-Bernal J.J., Araque A.F., García A.C., Fernandez-Lazaro C.I. (2020). Electromyography: A simple and accessible tool to assess physical performance and health during hypoxia training. A systematic review. Sustainability.

[64] Outlaw J.J., Wilborn C.D., Smith-Ryan A.E., Hayward S.E., Urbina S.L., Taylor L.W., Foster C.A. (2014). Acute effects of a commercially-available pre-workout supplement on markers of training: A double-blind study. J. Int. Soc. Sports Nutr..

[65] Van R.T., Van K.P., Vanden B.E., Puype J., Lefere T., Hespel P. (2009). β-alanine improves sprint performance in endurance cycling. Med. Sci. Sports Exerc..

[66] Hoffman J., Ratamess N., Kang J., Mangine G., Faigenbaum A., Stout J. (2006). Effect of creatine and ß-alanine supplementation on performance and endocrine responses in strength/power athletes. Int. J. Sport Nutr. Exerc. Metab..

[67] Zoeller R.F., Stout J.R., O’kroy J.A., Torok D.J., Mielke M. (2007). Effects of 28 days of beta-alanine and creatine monohydrate supplementation on aerobic power, ventilatory and lactate thresholds, and time to exhaustion. Amino Acids.

[68] Bassit R.A., da Justa Pinheiro C.H., Vitzel K.F., Sproesser A.J., Silveira L.R., Curi R. (2010). Effect of short-term creatine supplementation on markers of skeletal muscle damage after strenuous contractile activity. Eur. J. Appl. Physiol..

[69] Larsen F.J., Schiffer T.A., Borniquel S., Sahlin K., Ekblom B., Lundberg J.O., Weitzberg E. (2011). Dietary inorganic nitrate improves mitochondrial efficiency in humans. Cell Metab..

[70] Bernardo D.N.D., Bryk F.F., Fucs P.M. (2015). Influence of nitric oxide in the improvement of muscle power. Acta Ortop. Bras..

[71] Sureda A., Córdova A., Ferrer M.D., Pérez G., Tur J.A., Pons A. (2010). L-citrulline-malate influence over branched chain amino acid utilization during exercise. Eur. J. Appl. Physiol..

[72] Collier S.R., Casey D.P., Kanaley J.A. (2005). Growth hormone responses to varying doses of oral arginine. Growth Horm. IGF Res..

[73] Kanaley J.A. (2008). Growth hormone, arginine and exercise. Curr. Opin. Clin. Nutr. Metab. Care.

[74] Zajac A., Poprzecki S., Zebrowska A., Chalimoniuk M., Langfort J. (2010). Arginine and ornithine supplementation increases growth hormone and insulin-like growth factor-1 serum levels after heavy-resistance exercise in strength-trained athletes. J. Strength Cond. Res..

[75] Okovityi S.V., Shustov E.B. (2020). Ornitine-dependent mechanisms of muscle fatigue correction and recovery from physical activity. Vopr. Kurortol. Fizioter. Lech. Fiz. Kult..

[76] Eto B., Mod GLe Porquet D., Peres G. (1995). Glutamate-arginine salts and hormonal responses to exercise. Arch. Physiol. Biochem..

[77] Coqueiro A.Y., Rogero M.M., Tirapegui J. (2019). Glutamine as an anti-fatigue amino acid in sports nutrition. Nutrients.

[78] Nakhostin-Roohi B., Javanamani R., Zardoost N., Ramazanzadeh R. (2016). Influence of glutamine supplementation on muscle damage and oxidative stress indices following 14 km running. Hormozgan Med. J..

[79] Chen Y.-M., Li H., Chiu Y.-S., Huang C.-C., Chen W.-C. (2020). Supplementation of L-Arginine, L-Glutamine, Vitamin C, Vitamin E, folic acid, and green tea extract enhances serum nitric oxide content and antifatigue activity in mice. Evid.-Based Complement. Alternat. Med..

[80] Sutton E.E., Coll M.R., Deuster P.A. (2005). Ingestion of tyrosine: Effects on endurance, muscle strength, and anaerobic performance. Int. J. Sport Nutr. Exerc. Metab..

[81] Australian Institute of Sport Supplements and Sports Foods in High Performance Sport. https://www.ais.gov.au/__data/assets/pdf_file/0014/1000841/Position-Statement-Supplements-and-Sports-Foods-abridged_v2.pdf.

[82] Fernández-Lázaro D. (2019). Biological and molecular bases in the development of pathogenesis in multiple myeloma disease. Investig. Clin..

[83] Fernández-Lázaro D., Fernandez-Lazaro C.I., Caballero-García A., Córdova Martínez A. (2018). Immunomodulatory agents (IMiDs): Tools for multiple myeloma treatment. Rev. Med. Chil..

